# A newly-synthesized compound CP-07 alleviates microglia-mediated neuroinflammation and ischemic brain injury via inhibiting STAT3 phosphorylation

**DOI:** 10.2478/jtim-2023-0090

**Published:** 2023-07-05

**Authors:** Mengdi Guo, Qian Cao, Shengnan Xia, Xiang Cao, Jian Chen, Yi Qian, Xinyu Bao, Yun Xu

**Affiliations:** Department of Neurology, Nanjing Drum Tower Hospital, Affiliated Hospital of Medical School, Nanjing University, Nanjing 210008, Jiangsu Province, China; Jiangsu Key Laboratory for Molecular Medicine, Medical School of Nanjing University, Nanjing 210008, Jiangsu Province, China; Jiangsu Province Stroke Center for Diagnosis and Therapy, Nanjing 210008, Jiangsu Province, China;; Nanjing Neuropsychiatry Clinic Medical Center, Nanjing 210008, Jiangsu Province, China; Nanjing Drum Tower Hospital Clinical College of Traditional Chinese and Western Medicine, Nanjing University of Chinese Medicine, Nanjing 210008, Jiangsu Province, China

**Keywords:** Ischemic stroke, microglia, neuroinflammation, anti-inflammatory therapy, STAT3

## Abstract

**Background and Objectives:**

Overactivated glial cells, especially microglia, are core components in the progression of pathologic neuroinflammation, and the application of anti-inflammatory reagents has been regarded as a potential therapy in the management of infarction/reperfusion (I/R) brain injury. This research aims to clarify the anti-inflammatory efect of a novel lipophilic compound N-(2-[4-tert-butylphenyl]-2-[pyrrolidine-1-yl]ethyl)-7-methyl-4-oxo-4H-chromene-2-carboxamide (named CP-07 in this study) in LPS-stimulated BV2 cell line and primary mouse microglia, and its therapeutic effect on I/R brain injury.

**Method:**

Cell Counting Kit-8 assay was used to determine the maximal nontoxic dose of CP-07. The mRNA levels of representative proinflammatory cytokines were determined by quantitative real-time polymerase chain reaction both *in vitro* and *in vivo*. TTC staining was performed to calculate infarct volumes while behavioral tests were used to assess the neurological deficits at 24 h after middle cerebral artery occlusion (MCAO). Flow cytometry analysis and immunofluorescence staining were performed to calculate the percentage of pro-inflammatory microglia *in vivo*.A selective JAK2/STAT3 pathway inhibitor, AG490 was used to block STAT3 phosphorylation before the CP-07 anti-inflammation tests *in vitro*.

**Results:**

CP-07 could effectively suppress the mRNA levels of IL-6, IL-1β, iNOS and TNF-α induced by lipopolysaccharide (LPS) *in vitro*, and markedly block the evaluation of the fluorescence intensity of Iba-1 in primary mouse microglia. In middle cerebral arteryocclusion models, intraperitoneal injection with 1 mg/kg CP-07 significantly reduced cerebral infarct volumes at 24 h after surgery compared with vehicle treatment group, and promoted the recovery of neurological functions in MCAO mice. Further studies validated that CP-07 administration reduced the percentage of CD86 positive microglia after I/R injury, and the expression level of p-STAT3 was also markedly reduced in both microglial cells and the penumbra tissues. Blocking STAT3 phosphorylation with AG490 could completely eliminate the anti-inflammatory effects of CP-07, at least *in vitro*.

**Conclusion:**

We showed that a newly synthesized compound, CP-07, could effectively reduce the inflammatory responses in LPS-stimulated BV2 cells and primary mouse microglia, and overproduction of cytokines in middle cerebral artery occlusion mouse models by inhibiting STAT3 phosphorylation, leading to a neuroprotective effect on I/R brain injury.

## Introduction

Stroke is a major cause of long-term disability, cognitive decline and preventable death in adults worldwide.^[1]^Among all stroke cases, ischemic stroke accounts for approximately ninety percent, while thrombolysis and thrombectomy represent the first-line therapies for the treatment of ischemic stroke.^[2]^ Although vascular recanalization has dramatically improved disease outcomes, only a small portion of stroke patients can benefit from recanalization therapies because of the limited therapeutic time window as well as a series of side effects following reperfusion.^[3, 4]^ Thus, there is a rising need for new treatment options targeting critical pathologic mechanisms of ischemic stroke.

While primary neuronal cell death occurs a few minutes after the interruption of cerebral blood flow and is often irreversible, secondary brain injury, such as neuroinflammation triggered by damage-associated molecular patterns (DAMPs) from injured cells, is considered a remediable process.^[5]^ As brain-resident myeloid cells, microglia can serve critical functions in maintaining cerebral immune surveillance and respond quickly to the stimulation of danger signals. Previous studies have shown that microglia-mediated neuroinflammation plays a central role in proinflammatory reactions after cerebral ischemia.^[6]^ Overactivated microglia can undergo morphologic and functional transitions and release cytokines into the extracellular space following cerebral ischemia, where they bind cognate receptors and kill neuronal cells directly.^[7]^ They also influence the inflammatory microenvironment by modulating astrocyte reactivity and recruiting peripheral immune cells into the brain parenchyma, ultimately controlling the inflammatory cascade following infarction/ reperfusion (I/R) brain injury.^[8, 9]^ Thus, microglia-mediated neuroinflammation might serve as one of the most valuable therapeutic targets for stroke treatment.

Although a multitude of animal studies have demonstrated the potential of anti-inflammatory treatments to reduce infarct volume and ameliorate behavioural performance,^[10, 11]^ few identified molecular targets can be successfully developed into therapeutic reagents. In addition, some practical problems, such as systematic side effects and the poor permeability of the blood–brain barrier, limit the clinical application of existing anti-inflammatory drugs in stroke treatment. Developing new compounds with high druggability and safety will meet the pressing clinical needs for the translation of anti-inflammatory therapies. In the present study, as part of our ongoing studies committed to exploring novel pharmacotherapeutic strategies to alleviate poststroke neuroinflammation,^[12, 13]^ we tested the anti-inflammatory effect of a novel lipophilic compound N-(2-[4-tert-butylphenyl]-2-[pyrrolidine-1-yl]ethyl)-7-methyl-4-oxo-4H-chromene-2-carboxamide (named CP-07 in this study) in LPS-stimulated BV2 cell line and primary mouse microglia. We also evaluated its pharmacological effects on proinflammatory cytokine release and microglial phenotype switching, as well as its therapeutic actions in reducing infarct volumes and alleviating neurological deficits in MCAO mouse models.

## Materials and Methods

### Animals

Eight- to 9-week-old male C57BL/6J mice were procured from the Model Animal Research Center (MARC) of Nanjing University. Animals were maintained in a sterile condition, and provided with chow standard diets and clean water. All animal experiments were approved by the Institutional Animal Care and Use Committee of Nanjing University (2019AE01073).

### Middle cerebral artery occlusion models and compound administration

Mice were divided into two groups in terms of their body weights and intraperitoneally injected with vehicle or CP-07 (1 mg/ kg) 30 min before surgery onset. Transient middle cerebral artery occlusion (MCAO) models were induced as previously reported.^[11]^ Cerebral blood flow (CBF) was monitored by a doppler flowmetry (Perimed corporation, Stockholm, Sweden) and a decline of ipsilateral CBF > 30% confirmed the interruption of blood flow in the middle cerebral artery. Reperfusion was conducted after 50 min by withdrawal of the suture.

### Behavior tests

Modified neurological severity score (mNSS) system, forelimb grip test and foot fault test were used to assess the neurological deficits at 24 h after MCAO. All behavior tests were conducted by a researcher blinded to the group allocation. The mNSS system contained comprehensive assessments of motor function, sensory function, reflexes and balance, with a higher score indicating more severe symptoms. Muscular strength was evaluated by the forelimb grip test according to previous studies. Mice was suspended by the tail and the ability to hang onto a horizontal metal bar was evaluated using a grip strength meter (Bioseb, Chaville, France). For the foot fault test, mice were placed on an evaluated grid for 2 min, and the numbers of forelimb misplacements were counted during locomotion.

### 2,3,5-triphenyltetrazolium chloride (TTC) staining and sample collection

Mice were sacrificed by isoflurane inhalation, and the brains were immediately cut into six 1mm-thick serial slices in the coronal plane, and incubated with 2% TTC (Sigma-Aldrich, Saint Louis, USA) for 20 min to distinguish the necrotic tissues (white part) and living tissues (red part). Images were collected with a camera and analyzed by Image J (NIH, Bethesda, USA). The cerebral infarct volume was calculated from the sum of infarcted areas in all six brain sections. Ischemic penumbra tissues recognized as cortical red/white boundary were harvested immediately after image acquisition for further use.

### Cell culture and treatment

Primary mouse microglia cultures were established from the cortex of neonatal 1-day-old C57BL/6J mice as previously described. Briefly, mice were euthanized using CO^2^ asphyxiation and the brain tissues were quickly dissected in ice-cold saline. Brain membrane was removed and cerebral cortex was subjected to enzymatic digestion using 0.25% Trypsin-EDTA for 10 min. Cells were centrifuged at 800 r/ min for 10 min, and were maintained in T75 flasks (Corning) for 10–12 days. To separate microglia from mixed glial cultures, flasks were shaken at 250 r/ min for 15 min to minimize content of astrocyte. Microglia floating in the suspension were replanted into 6 well plates at a most suitable density. For stimulation, cells were pre-treated with or without AG490 for 1 h, then incubated with vehicle or CP-07 for 1 h, and 100 ng/mL LPS (Sigma-Aldrich, Saint Louis, USA) was added into the medium. Cells were harvested at 3 h or 12 h after LPS stimulation and culture supernatants were reserved at –80°C until use.

### Cell viability assay

BV2 or purified primary mouse microglia were cultured in 96 well plates at a density of 1 × 10^4^ cells/well and pretreated with CP-07 or vehicle for 24 h. Cell viability was measured with cell counting kit-8 (CCK-8; Abcam, Cambridge, USA). A microplate reader (Tecan, USA) was used to detect the OD values at 450 nm. Percentage of cell viability was calculated as follows: absorbance value of experimental group/absorbance value of control group × 100%.

### RNA extraction and quantitative real-time PCR

The total RNA of cell or tissue samples was isolated with TRIzol reagent (Invitrogen). Reverse transcription was performed by *Evo M-MLV* RT Kit (Accurate Biology). SYBR Green Reagent (Accurate Biology) and ABI 7500 PCR instrument (Applied Biosystems) were used for quantitative RT-PCR. Gene expression was normalized against GAPDH mRNA level. Primer sequences used in the current study were shown as Table 1.

**Table 1:** Primer sequences used in the current study.

Gene name	Forward primer	Reverse primer
*IL-1β*	5’-CTCACAAGCAGAGCACAAGC-3’	5’-CAGTCCAGCCCATACTTTAGG-3’
*IL-6*	5’-GGCGGATCGGATGTTGTGAT-3’	5’-GGACCCCAGACAATCGGTTG-3’
*iNOS*	5’-CAAGCACCTTGGAAGAGGAG-3’	5’-AAGGCCAAACACAGCATACC-3’
*TNF-α*	5’-CCCTCACACTCAGATCATCTTCT-3’	5’-GCTACGACGTGGGCTACAG-3’
*GAPDH*	5’-GCCAAGGCTGTGGGCAAGGT-3’	5’-TCTCCAGGCGGCACGTCAGA-3’

IL: interleukin; iNOS: inducible nitric oxide synthase; TNF-α: tumor necrosis factor α.

### Flow cytometry

Mice brain were harvested immediately after behavioral tests to obtain single cell suspension as previously described. Cells were resuspended in staining buffer (BD Biosciences, San Jose, USA), and incubated with Flow cytometry blocking buffer (BD Biosciences, San Jose, USA)) for 15 min. Subsequently, cells were washed two times and incubated with CD11b (53-0112-82, Invitrogen, CA, USA) and CD45 (25-0451-82, Invitrogen, CA, USA), and sorted cell population was identified as CD45^int^CD11b^+^. Then the sorted cells were incubated with CD86 (564198, BD Biosciences, San Jose, USA) , followed by thorough washing and finally centrifuged and resuspended in staining buffer for FACS analysis (BD Biosciences, San Jose, USA). Data analysis was performed using FlowJo software.

### Immunofluorescence staining

Mice were euthanized and trans-cardially perfused with 1×PBS (Solarbio, Beijing, China) and 4% PFA (pH 7.4) successively. Brain was quickly harvested and fixed with 4% PFA (Solarbio, Beijing, China), and dehydrated using 30% sucrose (Solarbio, Beijing, China) solution for at least 48h. Brains were then blotted dry and frozen at -80°C before brain sections (20 μmol/L) were prepared. For immunofluorescence staining, sections were permeabilized using 0.1% Triton X-100 (Aladdin, Shanghai, China) for 15–20 min, blocked in 2% bovine serum albumin (Aladdin, Shanghai, China) for 1–2 h, and incubated overnight with indicated antibodies: Iba-1 (ab178846, Abcam, Cambridge, USA, 1:500), p-STAT3 (#9145, Cell Signaling Technology, Beverly, USA, 1:200). Brain sections were incubated with fluorescence labeled secondary antibody (Alexa Fluor 488, 594, Invitrogen, CA, USA) for 2 h in the dark on the next day. Cell nuclei were stained with DAPI (Beyotime Biotechnology, Shanghai, China) for 10 min. Images were obtained with a confocal laser-scanning microscope (Olympus, Tokyo, Japan), and the fluorescence intensity were analyzed by Image J (NIH, Bethesda, USA).

**Figure 1:**
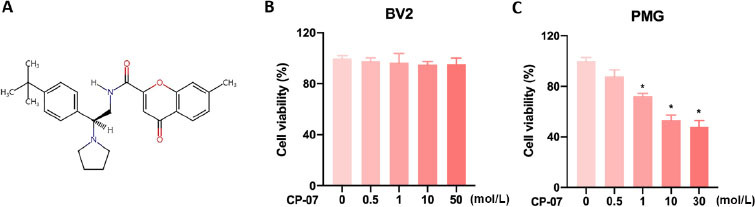
Cellular toxicity of CP-07 on BV2 cells and primary microglia. (A) Chemical structure of N-(2-[4-tert-butylphenyl]-2-[pyrrolidine-1-yl] ethyl)-7-methyl-4-oxo-4H-chromene-2-carboxamide (CP-07). (B) The viability of BV2 and primary mouse microglia after treated with various concentrations of CP-07 for 24 h was evaluated using cell counting kit-8 (CCK-8) assays. Values are presented as mean ± SEM in each group of four to six independent experimental procedures. *P < 0.001 *versus* 0 μmol/L group. PMG: primary mouse microglia.

### Western blotting

Tissues and cell samples were homogenized with RIPA lysis and extraction buffer containing 1% protease and phosphatase inhibitor cocktail (ThermoFisher, Waltham, USA). Protein quantification was performed with a BCA kit (ThermoFisher, Waltham, USA) as suggested by the manufacturer, and the optical densities were measured with a plate reader at 562 nm. For electrophoresis, protein samples were loaded onto 7.5%-12.5% SDS-PAGE gels (Sigma-Aldrich, Saint Louis, USA), transferred to PVDF membrane and blocked by 5% milk powder dissolved in 1×TBST to minimize the non-specific binding. Blotting was then detected with rabbit anti-STAT3, rabbit anti-p-STAT3 (#9145, Cell Signaling Technology, Beverly, USA), and β-actin (Bioworld Technology, Nanjing, China), followed by incubated with HRP–conjugated secondary antibodies (Bioworld Technology, Nanjing, China), and visualized by treating with ECL (MerckMillipore, Bedford, USA) and scanning with a Gel-Pro system (Tanon, Shanghai, China). Gray scale value of each protein band was analyzed using Image J (NIH, Bethesda, USA).

### Statistical analysis

All statistical analysis was done with the GraphPad Prism 9 (GraphPad Software, CA, USA). P value was calculated with Student’s t test or one-way ANOVA as appropriate. Statistical significance was indicated as *P* < 0.05.

## Results

### Cellular toxicity of CP-07 on BV2 cells and primary microglia

To evaluate the cellular toxicity of CP-07 (Figure 1A), BV2 cell lines and primary mouse microglia were seeded in 96-well plates and incubated with CP-07 at different concentration gradients for 24 h. CCK-8 assays were performed to assess the cellular toxicity and/ or growth suppression effects. As shown in Figure 1B, while BV2 cells presented excellent toxicity resistance to CP-07 even at a final concentration of 50 μmol/L, primary microglia were much more sensitive to the maximal nontoxic dose at 0.5 μmol/L (Figure 1C). Therefore, to further investigate the pharmacological activity adequately and minimize the effects of cell death, 0.5 μmol/L CP-07 was used in all the following *in vitro* experiments.

### Effects of CP-07 on inflammatory responses in LPS stimulated BV2 cells and primary microglia

**Figure 2:**
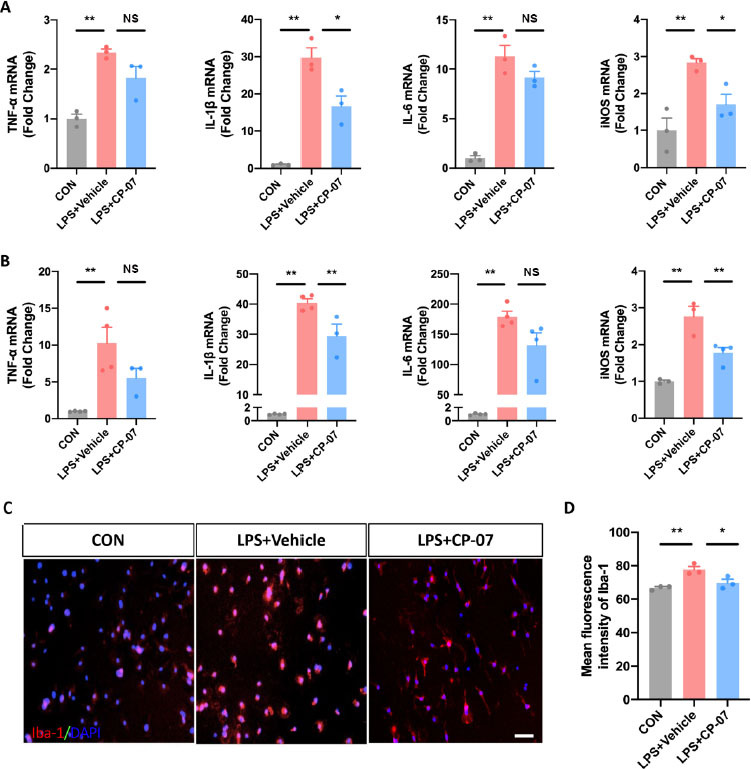
Effects of CP-07 on inflammatory responses in LPS stimulated BV2 cells and primary microglia. (A) Relative mRNA expression levels of Iba-1 and four pro-inflammatory mediators in BV2 cells were detected with RT-qPCR. (B) Relative mRNA levels of four inflammatory mediators from cultured primary mouse microglia. (C) PMG was immuno-stained for Iba-1 (red) and DAPI (blue) to identify microglia and the cell nuclei in different groups. (D) Quantitative analysis of mean fluorescence intensity of Iba-1. Scale bar: 50 μmol/L. Values are expressed as mean ± SEM of at least three independent experiments in each group, unless otherwise stated. NS, no significance. *P < 0.05 and **P < 0.01, *versus* LPS + Vehicle group. CON: control group; LPS: lipopolysaccharide treated group.

Next, we assessed the *in vitro* effects of CP-07 on microglia-mediated inflammatory responses. We first detected the mRNA levels of representative proinflammatory cytokines, such as interleukin (IL)-6, IL-1β, inducible nitric oxide synthase (iNOS) and tumor necrosis factor (TNF)-α, in the LPS-stimulated BV2 microglial cell line with or without CP-07. As shown in Figure 2A, the transcriptional levels of the above genes were significantly upregulated after LPS stimulation and decreased to a viable degree with CP-07 pretreatment. We further tested the anti-inflammatory effects of CP-07 in *in vitro* cultures of primary mouse microglia, which provided further proof of the anti-inflammatory potential of CP-07. The results of RT-qPCR indicated that CP-07 treatment could alleviate the overproduction of proinflammatory cytokines in primary mouse microglia, similar to BV2 cells (Figure 2B). Immunofluorescence staining also demonstrated that LPS stimulation induced a significant upregulation of the fluorescence intensity of Iba-1, while pretreatment with CP-07 markedly blocked this elevation (Figure 2C and D). Collectively, our data demonstrated that CP-07 could effectively suppress microglial inflammatory responses induced by LPS *in vitro*, which suggested that it may have therapeutic effects on neuroinflammation-related diseases.

### CP-7 alleviated ischemic brain injury in experimental stroke mice

**Figure 3:**
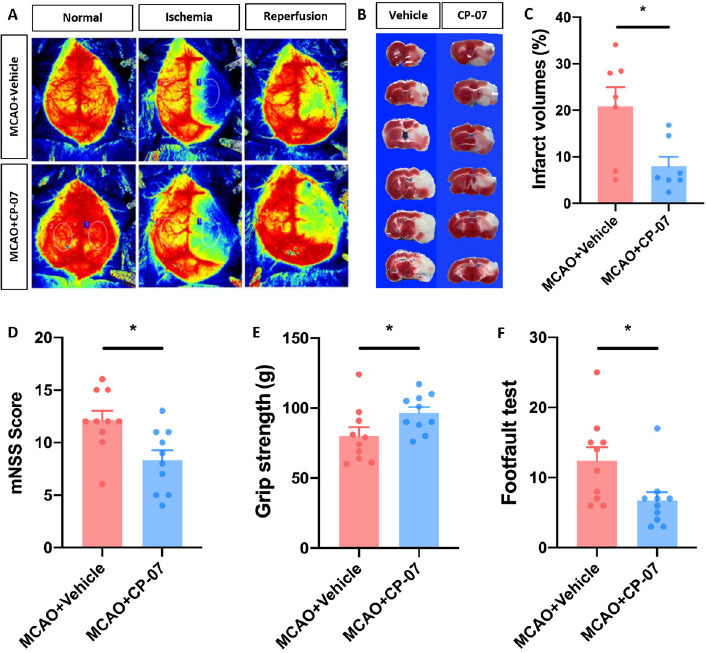
CP-7 alleviated I/R brain injury in experimental stroke mice. Vehicle or CP-07 was administrated to mice 30min before MCAO. (A) Laser-Doppler was used to measure CBF both in the resting state and ischemia/reperfusion stage. (B) TTC staining was performed to calculate infarct volumes, and (C) results were analyzed by Image J (*N* = 7). The neurological function of MCAO mice treated with vehicle or CP-07 was assessed by (D) mNSS, (E) grip strength and (F) footfault test (*N* = 10). Values are expressed as mean ± SEM in each group. *P < 0.05 *versus* MCAO + Vehicle group. MCAO: middle cerebral artery occlusion.

Since overactivated neuroinflammation has been considered a principal factor in the poor prognosis of ischemic stroke patients, we used the MCAO mouse model to determine whether CP-07 could alleviate ischemic brain injury. Eight-to 9-week-old male C57BL/6J mice subjected to MCAO operation were randomly divided into two groups. Body weight was not different between the two groups (data not shown). Each mouse was intraperitoneally injected with 1 mg/kg CP-07 or an equal volume of vehicle 30 min before surgery onset. Laser-Doppler analysis indicated that CP-07 did not influence CBF in either the resting state or the ischemia/reperfusion stage (Figure 3A and B). Neurological functions were assessed with a modified neurological severity score (mNSS) system, a forelimb grip test and a foot fault test 1 day after MCAO. Our data demonstrated that neurological deficits following cerebral I/R injury could be significantly alleviated after CP-07 treatment, as shown by the lower mNSS scores, enhanced forelimb grip strength and decreased foot fault (Figure 3D–F). Mice were sacrificed immediately after behavioural tests, and TTC staining was performed to evaluate infarct volumes. Compared with vehicle treatment, CP-07 administration significantly reduced cerebral infarct volumes 1 day after MCAO (Figure 3C). These results indicated that CP-07 administration promoted the recovery of neurological functions in MCAO mice and might serve as an efficient therapeutic strategy for ischemic brain injury.

### CP-07 reduced inflammatory responses of brain parenchyma following ischemic stroke

**Figure 4:**
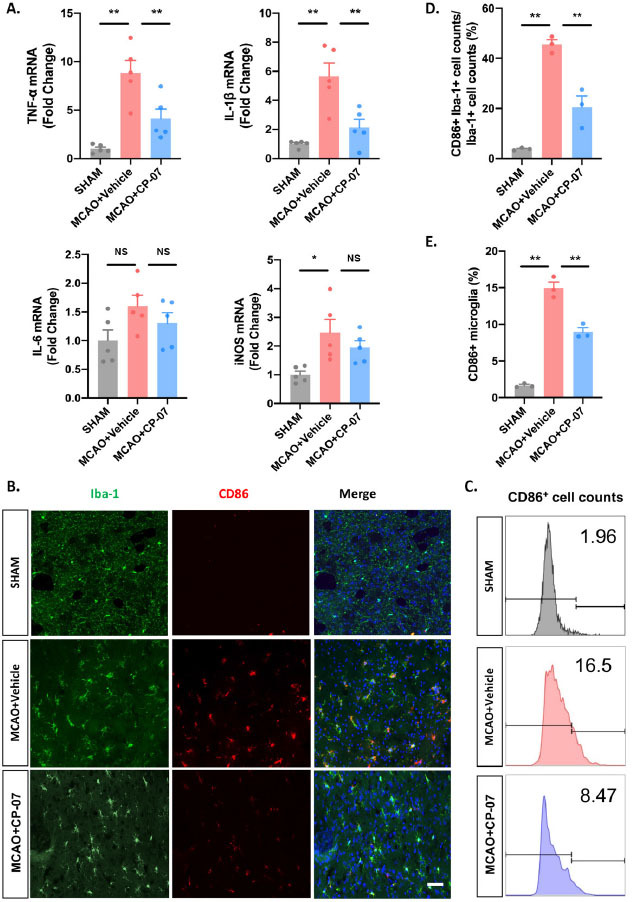
CP-07 reduced the inflammatory responses of brain parenchyma following ischemic stroke. (A) Tissues from the penumbra of ischemic hemisphere were harvested to detect the transcriptional levels of four pro-inflammatory cytokines. (B) Immunofluorescence staining for Iba-1 (green) and CD86 (red). (C) The proportion of CD86+Iba-1+ cell counts to the total Iba-1+ cell counts in different groups. (D) Flow cytometry analysis was performed to calculate the percentage of pro-inflammatory microglia. (E) Relative proportion of CD86+ microglial cells in different groups. Scale bar: 50 μmol/L. Values are expressed as mean ± SEM of at least three independent experiments in each group, unless otherwise stated. NS, no significance. *P < 0.05 and ^**^P < 0.01, *versus* MCAO + Vehicle group. SHAM: sham-operated animals; MCAO: middle cerebral artery occlusion.

Neuroinflammation is an important pathophysiology during ischemic stroke. As CP-07 showed potent anti-inflammatory function *in vitro* and alleviated ischemic brain injury, we further investigated whether it played a neuroprotective function in ischemic stroke by alleviating inflammatory responses of brain parenchyma. Tissues from the penumbra of the ischemic hemisphere were harvested for RT–qPCR analysis. As expected, the transcriptional levels of four proinflammatory cytokines were markedly decreased in CP-07-pretreated MCAO mice compared with those in the vehicle-treated group, which was consistent with the *in vitro* results (Figure 4A). Immunostaining of CD86 and Iba-1 indicated that the injection of CP-07 reduced the percentage of proinflammatory (indicated as CD86- and Iba-1-double-positive cells) microglia after I/ R injury (Figure 4B and D). Flow cytometry analysis also showed that CP-7 induced a significant decline in the number of CD86-positive microglia (Figure 4C and E), indicating that CP-07 could modulate the proinflammatory microglial phenotype switch following experimental I/R brain injury. Collectively, these results established reliable evidence that CP-07 could alleviate inflammatory responses of the brain parenchyma after ischemic stroke.

### Microglial STAT3 phosphorylation were significantly inhibited after CP-07 treatment both in vitro and in vivo

**Figure 5:**
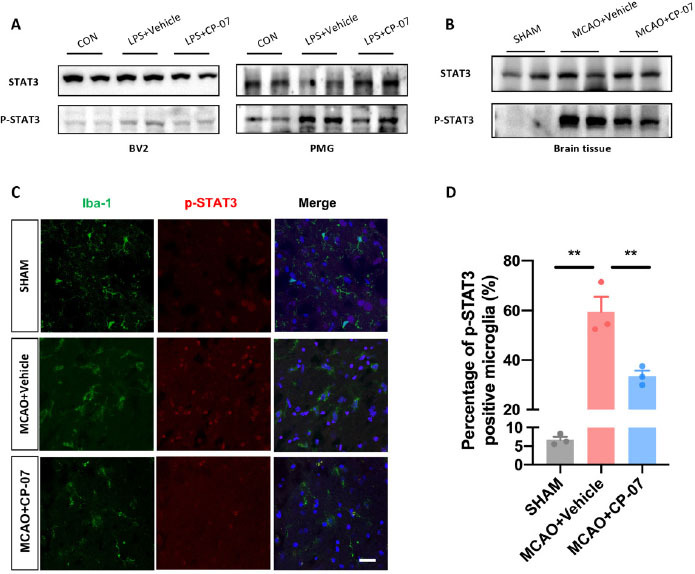
STAT3 phosphorylation were significantly inhibited after CP-07 treatment *in vivo*. (A) Western blotting results revealed that CP-07 could significantly inhibit STAT3 phosphorylation in LPS stimulated BV2 cells and primary mouse microglia. (B)Western blotting analysis of STAT3/phosphorylated STAT3 levels of brain tissues from sham, MCAO + vehicle and MCAO + CP-07 groups. (C) Immunofluorescence co-staining of Iba-1 (green) and p-STAT3 (red) was performed to reveal the influence of CP-07 on the phosphorylation of STAT3. Scale bar: 20 μmol/L. *N* = three slices from three mice in different groups. (D) Quantitative analysis of percentage of p-STAT3 positive microglia. Values are expressed as mean ± SEM for three mice in each group. **P < 0.01 *versus* MCAO + Vehicle group. CON: control group; LPS: lipopolysaccharide treated group; SHAM: sham-operated animals; MCAO: middle cerebral artery occlusion; PMG: primary mouse microglia.

In the following section, we conducted a preliminary exploration of the pharmacological mechanisms underlying the therapeutic effects of CP-07. Western blotting results revealed that CP-07 could significantly inhibit the phosphorylation level of STAT3 in both LPS-stimulated BV2 cells and primary mouse microglia (Figure 5A). We next investigated the relationship between the therapeutic effects of CP-07 and microglial STAT3 phosphorylation *in vivo*. Western blotting analysis demonstrated that the expression level of p-STAT3 was markedly reduced in the penumbra after CP-07 treatment (Figure 5B). In addition, immunofluorescence staining demonstrated that the number of p-STAT3-positive microglia was markedly reduced after CP-07 treatment one day after MCAO (Figure 5C and D). Taken together, these data explicitly showed that the protective effects of CP-07 in MCAO mice were at least partly related to the inhibition of microglial STAT3 phosphorylation.

### The selective p-STAT3 inhibitor AG490 eliminated the anti-inflammatory effects of CP-07 in vitro

**Figure 6:**
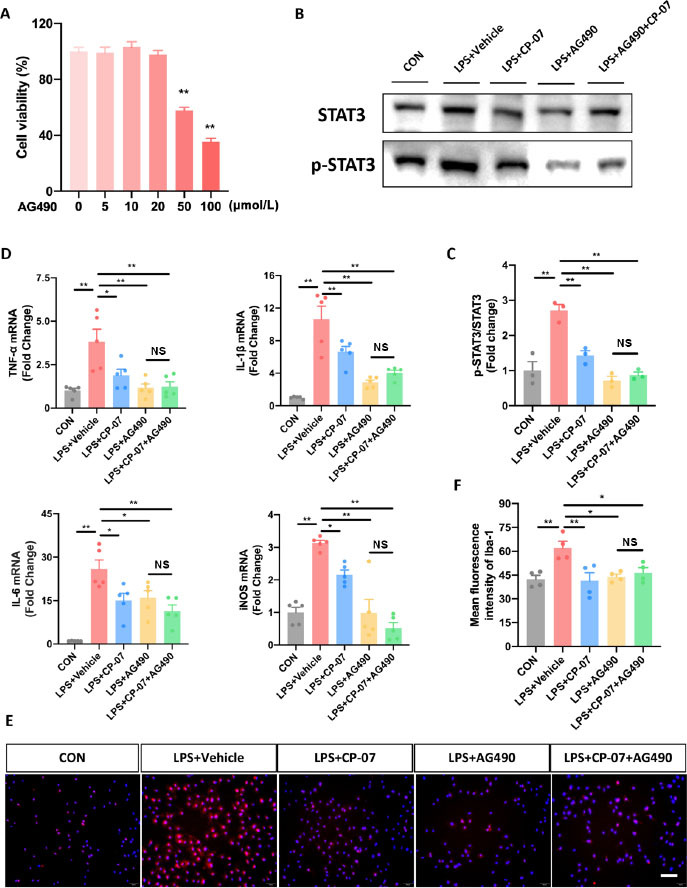
Selective p-STAT3 inhibitor AG490 eliminated the anti-inflammatory effects of CP-07 in vitro. (A) The viability of BV2 and PMG cells after treated with various concentrations of CP-07 for 24h was evaluated using CCK-8 assays. **P < 0.01, *versus* 0 μmol/L group. (B) Western blotting analysis of STAT3 and phosphorylated STAT3 levels in BV2 cells from different groups. (C) Quantitative analysis of relative fold change of p-STAT3/STAT3 expression level. (D) Relative mRNA expression levels of four pro-inflammatory mediators in BV2 cells from different groups were detected with RT-qPCR. (E) PMG was immuno-stained for Iba-1 (red) and DAPI (blue) to identify microglia and the cell nuclei in different groups. (F) Quantitative analysis of mean fluorescence intensity of Iba-1. Scale bar: 50 μmol/L. Values are expressed as mean ± SEM of at least three independent experiments in each group, unless otherwise stated. ns, no significance. *P < 0.05 and ^**^P < 0.01, *versus* indicated group. CON: control group; LPS: lipopolysaccharide treated group; TNF: tumor necrosis factor; IL: interleukin-6.

To confirm that the proposed mechanism of action for CP-07 is through inhibiting microglial STAT3 phosphorylation, BV2 cell lines and PMG were pretreated with or without AG490, a well-recognized and widely used selective JAK2/STAT3 pathway inhibitor, to block STAT3 phosphorylation before the CP-07 anti-inflammation tests. As shown in Figure 6A, CCK-8 assays were performed to assess the cellular toxicity of AG490 to BV2 cells, and the maximal nontoxic dose (20 μmol/L) was used in all the following experiments to occlude STAT3 phosphorylation to the greatest extent. Western blotting results revealed that pretreatment with AG490 at a 20 μmol/L concentration with or without subsequent CP-07 treatment significantly inhibited the phosphorylation level of STAT3 in LPS-stimulated BV2 cells, and specifically, no significant differences were observed between the LPS+AG490 group and the LPS+CP-07+AG490 group (Figure 6B and C). This result indicated that pretreatment with AG490 effectively blocked the ability of CP-07 to inhibit microglial STAT3 phosphorylation. Next, we detected the mRNA levels of proinflammatory mediators mentioned in the original version of our manuscript from different groups with RT–qPCR. As shown in Figure 6D, CP-07 treatment reduced the mRNA levels of TNF-α, IL-1β, IL-6 and iNOS in LPS-stimulated BV2 cells, but this anti-inflammatory phenotype was completely eliminated under p-STAT3 blockade with AG490. We also used immunofluorescence staining to provide more evidence in the PMG, and similar conclusion was obtained from the LPS+AG490 and LPS+CP-07+AG490 groups (Figure 6E and F). In summary, these rescue experiments further verified our previous conclusion that CP-07 alleviated microglia-mediated neuroinflammation by inhibiting STAT3 phosphorylation, at least *in vitro*.

## Discussion

As a highly disabling and lethal disorder with limited therapeutic options, ischemic stroke affects millions of people around the world. Proinflammatory microglia have been considered the main pathogenic drivers of secondary neuronal death following ischemia/reperfusion brain injury,^[5]^ and numerous studies have demonstrated the destructive effects of excessive microglia-related inflammation on the long-term prognosis of stroke patients. However, there is insufficient evidence to suggest that therapeutic intervention targeting neuroinflammation can be beneficial, which may be due to the lack of efficient drugs, at least in part.^[14,15]^ In this study, we showed that a newly synthesized compound, CP-07, could effectively reduce the inflammatory responses in LPS-stimulated BV2 cells and primary mouse microglia, the proinflammatory microglial phenotype switch and overproduction of cytokines in middle cerebral artery occlusion mouse models by inhibiting STAT3 phosphorylation, ultimately leading to a neuroprotective effect on I/ R brain injury.

Microglia is the first immune cell to sense the imbalance of the cerebral microenvironment. They express various kinds of pathogen detectors, signalling sensors and neurotransmitter receptors for prompt responses under physiological and pathological conditions. Previous studies have demonstrated the detrimental effects of a proinflammatory microglial phenotype and certain inflammatory cytokines on the long-term prognosis of ischemic stroke. Hu *et al*. found that microglia responded dynamically to I/R brain injury and experienced a transition to a ‘sick’ M1 phenotype similar to macrophages in the early phase of cerebral ischemia.^[16,17]^ This microglial phenotype was identified as a proinflammatory and neurotoxic phenotype and expressed high levels of CD86, CD11b and iNOS. They also exacerbated oxygen glucose deprivation (OGD)-induced neuronal cell death *in vitro* and inflammatory responses following I/R brain injury *in vivo*. In the acute stage of ischemic stroke, microglia-derived proinflammatory cytokines have been identified as the main contributors to the expansion of infarct areas.^[6]^ They could be rapidly released by DAMP stimulation and promote the migration and proliferation of glial cells around peri-infarct regions. Circulating immune cells are also recruited into the brain parenchyma in the presence of microglia-derived chemokines, which are involved in communication with resident immune cells and participate in the overproduction of inflammatory cytokines.^[18]^ Among the classical inflammatory mediators, TNF-α, iNOS, IL-1β and IL-6 have been the most extensively studied. TNF-α was reported to be primarily released by microglia and peaked at 12–24 h following ischemic stroke, where it bound its cognate receptors and killed neuronal cells directly.^[11]^ iNOS was the main rate-limiting enzyme of nitric oxide production under inflammatory states and was generally known to be neurotoxic.^[19]^ IL-1β was also shown to aggravate neuroinflammation as an amplifier of the inflammatory cascade. Microglia-derived IL-1β triggered the proliferation and activation of astrocytes, leading to the production of VEGF-A and disruption of the BBB.^[20, 21]^ Regarding IL-6, its exact function in I/R brain injury was still controversial. Classic IL-6 signalling was proven to be neuroprotective, while trans-signalling showed proinflammatory effects and was responsible for the expansion of infarct areas.^[22, 23]^ In the present study, administration of CP-07 effectively modulated the proinflammatory microglial phenotype switch and overproduction of the above cytokines following MCAO, calling for further translational studies of anti-inflammatory strategies.

The phosphorylation state of STAT3 provides a molecular switch on the balance between homeostasis and inflammation. Inhibition of phosphorylation could prevent the subcellular translocation of STAT3 and decrease the expression of downstream cytokines in microglia, including IL-6 and TNF-α, at the transcriptional level. Many studies have widely confirmed that inhibition of STAT3 phosphorylation using genetic or pharmacologic approaches could significantly improve neurological outcomes in cerebral ischemic models,^[24, 25]^ raising hopes for new therapeutic modalities of stroke.^[26, 27]^ Recent studies have also explored the therapeutic effects of several direct or indirect p-STAT3 inhibitors in rodent models of acute brain injury.^[28, 29]^ Zhu *et al*.^[30]^ demonstrated that ruxolitinib alleviated ischemic brain injury by inhibiting the function of microglial JAK2, a key kinase upstream of STAT3, which downregulated microglial cytokine expression and promoted the ‘M2’ phenotype switch. Zheng *et al*.^[32]^ generated microglia-conditional STAT3 knockout mice using the Cre-LoxP system and clarified the role of STAT3 and the downstream pathways in microglia-mediated neuroinflammation after experimental subarachnoid haemorrhage.^[31]^ Moreover, p-STAT3 inhibition could reduce excessive glial scar formation, which cleared the major obstacle for neuronal repair and promoted functional recovery in the chronic stage of ischemic stroke.^[33]^ Even in some studies aiming at other therapeutic targets, modulation of the STAT3 signalling pathway might also be involved as the underlying mechanism. For example, Shan *et al*. investigated the therapeutic effect of exendin-4, a glucagon-like peptide-1 receptor (GLP-1R) agonist, on inflammation and BBB breakdown secondary to stroke and found that exendin-4 significantly reduced JAK2/STAT3 activation but not the activation of PI3K/Akt, suggesting a nonclassical signalling pathway downstream of GLP-1R.^[34]^ These results convincingly proved the central role of STAT3 phosphorylation in the pathologic activation of neuroinflammation and its druggability as a valuable therapeutic target for stroke treatment, which was consistent with the pharmacological effects of CP-07 verified in our present study.

Our study still has several limitations. First, although we have preliminarily demonstrated the anti-inflammatory effect of CP-07 in overactivated microglia both *in vitro* and *in vivo*, more studies are still needed to further investigate the effects of CP-07 on other cerebral cell types to uncover its detailed pharmacological mechanisms. Second, phosphorylated STAT3 may not be the direct target of CP-07. Since many factors could affect the phosphorylation state of STAT3 (such as the conformation of the STAT3 SH2 domain,^[35, 36]^ the activity of upstream kinases or the expression levels of cell-surface receptors^[37, 38]^), more experiments should be carried out to explicitly identify the real target protein of CP-07. Third, it should be noted that using two or more p-STAT3-specific inhibitors would help to reduce the off-target effects, and microglia-specific STAT3 knockout (KO) mice may provide the strongest proof and completely eliminate the off-target effects and residual expression of STAT3 caused by siRNA/ shRNA-based gene knockdown. ^[39, 40]^ These studies will make our conclusions more credible. As a final point, the delivery mode of CP-07 (intraperitoneal injection) and the time point of intervention (30 min before MCAO onset) used in this study lack clinical utility. Alternate routes and delayed time points of drug administration should also be evaluated in the future.

## Conclusion

In summary, we identified a newly synthesized compound, CP-07, which could reduce the inflammatory responses in LPS-stimulated BV2 and primary microglia, as well as the proinflammatory microglial phenotype switch and overproduction of cytokines in MCAO mouse models *via* inhibition of STAT3 phosphorylation. CP-07 showed satisfactory therapeutic effects in mice suffering ischemic stroke, leading to a reduction in infarct volume and an improvement in neurological function recovery. Since the microglia-mediated pathogenic inflammatory cascade in the brain parenchyma and the phosphorylation extent of STAT3 are major pathogenic drivers contributing to I/R brain injury, the discovery of CP-07 might have potential clinical utility for stroke treatment.
